# Association of Asthma with Acute Vaso-Occlusive Crisis Among French Guianese Children with Sickle Cell Disease

**DOI:** 10.1007/s12098-025-05749-7

**Published:** 2025-09-05

**Authors:** Gabriel Bafunyembaka, Mathieu Nacher, Chimène Maniassom, Archippe Birindwa, Narcisse Elenga

**Affiliations:** 1 Department of Pediatrics, Franck Joly Hospital, Western French Guiana, Avenue Paul Castaing Saint-Laurent du Maroni GF 97320, Guyane Française, French Guiana, France; 2 Clinical Investigation Center, Epidemiology/Public Health, Inserm 1424, Cayenne Hospital, Avenue Alexis Blaise 97300 Cayenne, French Guiana, France; 3 Department of Pediatric Medicine and Surgery, Cayenne Hospital, Avenue Alexis Blaise 97300 Cayenne, French Guiana, France; 4 Department of Pediatrics, Bukavu University Hospital, Official University of Bukavu, Bukavu, Democratic Republic of Congo; 5 Sickle Cell Disease Center, Cayenne Hospital, Avenue Alexis Blaise 97300 Cayenne, French Guiana, France

**Keywords:** Hospitalization, Vaso-occlusive crises, Sickle cell disease, Asthma

## Abstract

**Objectives:**

To evaluate the impact of asthma on hospitalizations for acute vaso-occlusive pain episodes in children with sickle cell disease (SCD).

**Methods:**

A multicenter nested case-control study was conducted over a period from January 1, 2012, to December 31, 2022.

**Results:**

The mean age of the study population was 8.41 ± 5.04 y, with an equal distribution of males and females. Among the 600 children with SCD included in the study, 35 were diagnosed with asthma, yielding a prevalence of 5.8%. Children with both SCD and asthma had a median of 6 hospitalizations (Range: 2–20), compared to 2 (Range: 1–4) in those without asthma (*p* < 0.001). In 90% of these cases, asthma exacerbation coincided with a sickle cell crisis. Besides vaso-occlusive episodes, other leading causes of hospitalization included pulmonary infections and acute chest syndrome. The incidence of acute chest syndrome was found to be 18 times higher in children with both asthma and sickle cell disease. Furthermore, children with both conditions were 3.99 times more likely to experience a vaso-occlusive crisis requiring hospitalization than those without asthma.

**Conclusions:**

Children with sickle cell disease and asthma co-morbidity experience a significantly higher number of vaso-occlusive crises requiring hospitalization.

## Introduction

Sickle cell disease (SCD) is the most widespread genetic disorder globally, affecting over 5 million individuals. It is the most common rare disease in France and constitutes a significant public health issue in French Guiana, which has the highest incidence rate among French overseas territories—estimated at 1/227 [1]. Asthma is a prevalent chronic respiratory condition characterized by airway inflammation and bronchoconstriction [2]. In the general pediatric population of French Guiana, asthma prevalence ranges from 10% to 16%. While the exact cause of asthma remains unknown, several risk factors have been identified, and gene-environment interactions are thought to play a crucial role. Asthma and SCD are two prevalent chronic diseases in French Guiana. SCD is an inherited condition, whereas asthma results from a complex interplay of genetic predisposition and environmental factors. Notably, the incidence of asthma among children with SCD is as high as 27%, which contributes to a lower quality of life in this population [3, 4]. Although the reason for the disproportionately high incidence of asthma in children with SCD is not yet fully understood, it is essential to explore its clinical implications to improve health outcomes in this vulnerable group. Several studies have reported that asthma is common in children with SCD and is associated with an increased risk of vaso-occlusive pain episodes, acute chest syndrome, and premature mortality [5–8]. However, most of these findings originate from developed countries with better living conditions.

Despite having the highest gross domestic product (GDP) per capita in Latin America and a largely free, universal healthcare system, French Guiana faces significant health disparities [9, 10]: approximately half of the population lives below the poverty line. These socioeconomic, cultural, environmental, and health-related factors may influence—or confound—the relationship between asthma and SCD. Thus, the context in French Guiana has a number of climatic, cultural, socioeconomic, and health specificities that may interact with or confound the relationship between asthma and SCD. Therefore, the objective of this study is to assess the impact of asthma on hospitalization rates for vaso-occlusive crises (VOC) among children with SCD in French Guiana.

## Material and Methods

This was a retrospective, multicenter nested case-control study of children with SCD diagnosed by universal neonatal screening, followed in French Guiana from January 1, 2012, to December 31, 2022. Of the 600 children followed, 35 were also asthmatic.

Asthma was defined in the medical records according to International Classification of Diseases 10th Revision (ICD-10, J45.9), supplemented by information in the medical records on the presence of at least one episode of wheeze not directly related to a viral upper respiratory tract infection requiring the prescription of bronchodilators or inhaled corticosteroids, or if the patient was treated with long-term inhaled corticosteroids.

Patients aged 6 mo to 18 y with a diagnosis of SCD (neonatal screening confirmed by genetic examination at six months of age) were selected from the computerized files. Data were extracted from the three main hospitals in French Guiana (Centre Hospitalier de Cayenne, CHC; Center Hospitalier de l’Ouest-Guyane Franck Joly, CHOG; and Center Hospitalier de Kourou, CHK).

Of the three areas, Cayenne was the most populated with 350 patients, followed by Saint-Laurent-du-Maroni with 200 patients and Kourou with 50 patients. In all three cities, the socio-economic conditions were similar, with 75% of children with sickle cell disease born to migrant parents. The quality of follow-up was defined by the annual number of consultations, and was poor for children who had not had a sickle cell consultation for more than a year. The quality of follow-up was similar in the three centres.

For each file, age, sex, place of residence, co-morbidity of asthma, quality of follow-up and the annual number of hospital admissions for acute vaso-occlusive pain episodes, based on ICD-10, were recorded. Quality of follow-up care was assessed based on the criterion of attending a minimum of three sickle cell consultations per year.

This study is classified as research not involving human subjects and falls under the “Reference Methodology” (MR-004), for which the CHC signed a compliance commitment on 25 September 2023. A privacy impact assessment was conducted, and a summary of the study has been published on the Health Data Hub website. The legal basis for data processing is the fulfillment of a public interest mission. The study was registered in the data processing register by the data protection officer of the three participating hospitals.

Patient consent was obtained *via* postal mail through an information sheet and a non-opposition form. In accordance with applicable regulatory provisions, the absence of a response within one month of mailing the non-opposition form was interpreted as a lack of objection.

Two groups - asthmatic and non-asthmatic children with SCD, were compared. The outcome was hospitalization for an acute vaso-occlusive pain episode. All analyses were performed using STATA 17 software (Stata Corp LP, College Station, TX, USA) [2]. To identify the determinants of hospitalization and the factors associated with hospitalization for acute vaso-occlusive pain episode, two simple and multiple linear regression models were constructed. As a measure of association, the unadjusted and adjusted beta coefficients with 95% confidence intervals are presented.

## Results

Among the 600 sickle cell cases collected, 35 children were diagnosed with asthma, corresponding to an estimated prevalence of 5.8% [95% confidence interval (CI): 4–8%]. There were 35 cases and 140 matched controls. No statistically significant differences were observed in the sociodemographic characteristics between asthmatic and non-asthmatic patients. The sociodemographic data of the study participants are presented in Table 1. Table 2 summarizes the frequency of hospitalizations for vaso-occlusive crises in the study groups.

**Table 1:** Sociodemographic characteristics of the study group

Variables	Number	Percentage (%)
Age (years)		
0–5	26	15.00
5–10	71	41.00
10–15	30	17.00
> 15	48	27.00
Gender		
F	85	48.57
M	90	51.43
Types of patients		
Sickle cell disease with asthma	35	20.00
Sickle cell disease without asthma	140	80.00
Place of residence		
Kourou	22	12.57
Cayenne	46	26.29
Saint-Laurent du Maroni	107	61.14
Resides with his familial unit		
No	22	12.57
Yes	153	87.47
Health insurance		
No	18	10.29
Yes	157	89.71
Sickle cell disease follow-up		
Regular	111	63.43
Irregular	64	36.57

**Table 2:** Frequency of hospitalizations for vaso-occlusive crises in study groups

Variables	Hospitalization frequency (Mean ± SD) or Median [IQR]	*P* value
Gender		0.501
F	3.12 ± 2.01	
M	3.32 ± 2.29	
Place of residence		0.484
Kourou	3 [1–12]	
Cayenne	3 [1–20]	
Saint-Laurent du Maroni	3 [1–09]	
Sickle cell disease follow-up quality		0.144
Regular	3 [0–10]	
Irregular	3 [1–20]	
Frequency of hospitalization		< 0.001
Non-asthmatic	2 [1–4]	
Asthmatic	6 [2–20]	

*IQR* Interquartile range; *SD* Standard deviation

Table 3 highlights the determinants of hospitalization in each group, with a statistically significant difference observed only in the asthmatic group (*p* < 0.001). Asthmatic sickle cell patients had a median of six annual hospitalizations, compared with two in non-asthmatic sickle cell patients. Eighty percent of children with asthma were hospitalized due to an acute exacerbation of their condition. In 90% of these cases, the exacerbation was associated with a sickle cell crisis. In addition to vaso-occlusive episodes, other leading causes of hospitalization included pulmonary infections and acute chest syndrome. The incidence of the latter was found to be 18 times higher in children with asthma or sickle cell disease compared to those without these conditions. Furthermore, children with SCD who also had asthma were 3.99 times more likely to experience a vaso-occlusive crisis requiring hospitalization compared to those without asthma.

**Table 3:** Determinants of hospitalization for vaso-occlusive crisis

Variables	Crude β (CI 95%)	*P* value	Adjusted β (CI 95%)	*P* value
Age	-0.007 (-0.06; 0.05)	0.796	-0.002 (-0.04; 0.04)	0.917
Gender				
F	Ref		Ref	
M	0.20 (-0.38; 0.78)	0.502	0.29 (-0.13; 0.73)	0.182
Frequency of hospitalization				
Non-asthmatic	Ref		Ref	
Asthmatic	4.01 (3.44; 4.58)	< 0.001	3.99 (3.39; 4.58)	< 0.001
Place of residence				
Kourou	1.04 (0.09; 1.98)	0.030	0.40 (-0.29; 1.10)	0.259
Cayenne	0.09 (-0.56; 0.74)	0.779	0.30 (-0.18; 0.79)	0.214
Saint-Laurent du Maroni	Ref		Ref	
Sickle cell disease follow-up quality				
Regular	Ref		Ref	
Irregular	0.74	0.016	0.01 (-0.45; 0.49)	0.08

*Adjusted β* Adjusted linear regression coefficient; *Crude β* Unadjusted linear regression coefficient; *Ref* Subjects less likely to be hospitalized for vaso-occlusive crisis

## Discussion

The estimated prevalence of asthma in children with SCD in the present study is notably lower than that reported in mainland France [11]. In the literature, estimates of asthma prevalence in children with SCD varies widely, ranging from 2% to 50% [12]. In the United States, prevalence estimates among African-American children range between 15% and 20% [8]. The lower prevalence in the present study may reflect underdiagnosis, likely due to the absence of universal asthma screening in these populations. Children exhibiting wheezing—a hallmark symptom of asthma—are typically referred to pediatric asthma specialists; however, until recently, there was only one pediatric asthma specialist per 105,000 children under 16 in authors’ region. This limited specialist availability likely contributed to underdiagnosis. Recent efforts, including the recruitment of a second pediatric asthma specialist and the implementation of systematic asthma screening for children with SCD, aim to address this gap.

The present study identified a significant association between asthma and hospitalization due to acute vaso-occlusive pain episodes in children with SCD. This relationship between co-morbid asthma and increased hospitalizations for vaso-occlusive episodes has been well documented in numerous studies [3–8]. The longest follow-up study on this topic, conducted by Boyd et al., demonstrated that a diagnosis of asthma was linked to an increased rate of pain episodes in children with SCD, compared to their non-asthmatic counterparts [8]. Indeed, pediatric patients with SCD and asthma have a significantly higher risk of hospitalization for severe pain episodes and experience twice the mortality rate compared to those without asthma [5]. Another study reported that children with SCD and asthma experienced nearly twice as many acute vaso-occlusive pain episodes as those without asthma [13]. Contrary to present findings, research on children with sickle cell anemia in France found no association between asthma and increased hospitalization rates for pain [14]. This discrepancy raises a critical question: how can we distinguish respiratory symptoms attributable to the progression of SCD from those indicative of concomitant asthma in children with SCD?

Although prior studies have reported varying asthma prevalence rates in SCD, a study by Angel et al. found no significant difference in asthma prevalence between patients with SCD and healthy controls [15]. Furthermore, children with SCD were less likely to present with wheezing, dry night cough, or use of corticosteroids compared to the control group. Interestingly, while children with SCD reported fewer asthma-related symptoms based on questionnaires, they experienced higher rates of hospitalization for respiratory illness in the previous 12 mo [15]. Asthma-like manifestations-including wheezing, chest tightness, and recurrent coughing-are common in pediatric patients with SCD, and airway hyperreactivity appears to be more prevalent in this population than in the general population, regardless of a formal asthma diagnosis [16–18]. The mechanisms underlying these asthma-like symptoms remain unclear, and there is ongoing debate as to whether they should be attributed solely to asthma or regarded as intrinsic features of SCD [4, 16]. Distinguishing between respiratory symptoms resulting from SCD progression and those indicative of co-morbid asthma in children with SCD remains a clinical challenge. Several key factors can aid in delineating this boundary: (i) Triggering factors: Asthma symptoms in children are typically precipitated by exposure to allergens, cold air, or physical exertion. In contrast, respiratory complications in SCD, such as acute chest syndrome (ACS), are more commonly associated with infections, fat embolism, or vaso-occlusive events; (ii) Response to treatment: Asthma-related symptoms generally respond favorably to bronchodilator therapy (e.g., short-acting β2-agonists), whereas pulmonary complications linked to SCD do not consistently improve with these agents. Notably, although bronchodilators may sometimes be used in the management of ACS, their effectiveness is variable and often limited to cases with co-existing reactive airway components; (iii) Pulmonary function testing: Spirometry can provide valuable diagnostic differentiation. Asthma is typically characterized by an obstructive ventilatory pattern, with reversibility post-bronchodilator. In contrast, children with SCD more frequently present with a restrictive ventilatory pattern, reflective of underlying parenchymal or pleural disease.

Together, these clinical, therapeutic, and functional parameters help refine the diagnostic approach, though overlap between the two conditions may occur. A careful longitudinal assessment is often required to accurately attribute respiratory symptoms to asthma vs. SCD-related pathology. Despite this controversy, hematologists caring for patients with SCD need to become familiar with asthma and develop a multidisciplinary approach, including early detection, appropriate management, and referral to asthma specialists.

The main limitation of present study was the small sample size. Furthermore, the authors were unable to establish a causal relationship between asthma exacerbation and acute vaso-occlusive pain episode occurrence in their patients. In addition, this was a retrospective study with probable bias in data collection. However, the authors were unable to obtain data on hydroxyurea (HU). They were unable to investigate the potential beneficial effects of HU on the frequency of acute vaso-occlusive pain episodes [19–21].

Despite these limitations, the new information provided by this study will enable clinicians in the region to pay particular attention to the optimal management and follow-up of children with sickle cell disease, especially those with asthma co-morbidity.

In summary, studies investigating the relationship between asthma and the rate of pain episodes in children with SCD have shown inconsistent results. However, in French Guiana, children with SCD and co-morbid asthma had an increased number of acute vaso-occlusive pain episodes requiring hospitalization. Therefore, the association between asthma and pain episodes should be considered preliminary until further evidence documents the association. With increasing recognition of the importance of asthma in SCD management, hematologists must become familiar with asthma and develop a multidisciplinary approach, including early detection by systematic screening, appropriate management, and referral to asthma specialists.

## Conclusions

In French Guiana, children with sickle cell disease and co-morbid asthma experienced an increased number of acute vaso-occlusive pain episodes requiring hospitalization. Given the inconsistencies reported in the literature, the present findings should be confirmed through prospective studies with larger sample sizes.

## References

[1] Etienne-Julan M, Muszlak M, Vaz T, Elenga N, Loko G, Elana G. Sickle cell disease in the French overseas departments (Antilles, French Guiana, Réunion, Mayotte). Descriptive data and organization of care [Article in French]. Wkly Epidemiol Bull (27–28, 03/07/2012).

[2] Miller RL, Grayson MH, Strothman K. Advances in asthma: new understandings of asthma’s natural history, risk factors, underlying mechanisms, and clinical management. J Allergy Clin Immunol. 2021;148:1430–41. 34655640 10.1016/j.jaci.2021.10.001

[3] An P, Barron-Casella EA, Strunk RC, Hamilton RG, Casella JF, Debaun MR. Elevation of IgE in children with sickle cell disease is associated with doctor diagnosis of asthma and increased morbidity. J Allergy Clin Immunol. 2011;127:1440–6. 21388662 10.1016/j.jaci.2010.12.1114 PMC3105194

[4] Indolfi C, Dinardo G, Grella C, et al. Exploring the interplay between asthma and hemoglobinopathies: A comprehensive review. J Clin Med. 2024;13:3263. 38892971 10.3390/jcm13113263 PMC11172992

[5] DeBaun MR, Strunk RC. The intersection between asthma and acute chest syndrome in children with sickle-cell anaemia. Lancet. 2016;387:2545–53. 27353685 10.1016/S0140-6736(16)00145-8 PMC5041533

[6] Knight-Madden JM, Forrester TS, Lewis NA, Greenough A. Asthma in children with sickle cell disease and its association with acute chest syndrome. Thorax. 2005;60:206–10. 15741436 10.1136/thx.2004.029165 PMC1747351

[7] Duckworth L, Black LV, Ezmigna D, et al. Spirometry use in patients with sickle cell disease with and without asthma and acute chest syndrome: A multicenter study. EJHaem. 2020;1:239–42. 32924025 10.1002/jha2.42 PMC7480828

[8] Boyd JH, Macklin EA, Strunk RC, DeBaun MR. Asthma is associated with acute chest syndrome and pain in children with sickle cell anemia. Blood. 2006;108:2923–7. 16690969 10.1182/blood-2006-01-011072 PMC1892235

[9] Van Melle A, Cropet C, Parriault MC, et al. Renouncing care in French Guiana: the National health barometer survey. BMC Health Serv Res. 2019;19:99. 30728033 10.1186/s12913-019-3895-6 PMC6366016

[10] Valmy L, Gontier B, Parriault MC, et al. Prevalence and predictive factors for renouncing medical care in poor populations of Cayenne, French Guiana. BMC Health Serv Res. 2016;16:34. 26822003 10.1186/s12913-016-1284-y PMC4731954

[11] Delmas MC, Guignon N, Leynaert B, et al. Prévalence et contrôle de l’asthme chez le jeune enfant en France [Prevalence and control of asthma in young children in France]. Rev Mal Respir. 2012;29:688–96. 22682595 10.1016/j.rmr.2011.11.016

[12] Bryant R. Asthma in the pediatric sickle cell patient with acute chest syndrome. J Pediatr Health Care. 2005;19:157–62. 15867831 10.1016/j.pedhc.2004.12.003

[13] Anim SO, Strunk RC, DeBaun MR. Asthma morbidity and treatment in children with sickle cell disease. Expert Rev Respir Med. 2011;5:635–45. 21955234 10.1586/ers.11.64 PMC3233260

[14] Bernaudin F, Strunk RC, Kamdem A, et al. Asthma is associated with acute chest syndrome, but not with an increased rate of hospitalization for pain among children in France with sickle cell anemia: A retrospective cohort study. Haematologica. 2008;93:1917–8. 18815195 10.3324/haematol.13090

[15] Angel A, Wandalsen GF, Solé D, et al. Asthma, allergic sensitization and lung function in sickle cell disease. Allergol Immunopathol. 2020;48:450–7. 10.1016/j.aller.2019.12.012 32249096

[16] Field JJ, DeBaun MR. Asthma and sickle cell disease: two distinct diseases or part of the same process? Hematol Am Soc Hematol Educ Program. 2009:45–53. 10.1182/asheducation-2009.1.45 20008181

[17] Glassberg JA, Strunk R, DeBaun MR. Wheezing in children with sickle cell disease. Curr Opin Pediatr. 2014;26:9–18. 24370489 10.1097/MOP.0000000000000045 PMC4167421

[18] Willen SM, Rodeghier M, DeBaun MR. Asthma in children with sickle cell disease. Curr Opin Pediatr. 2019;31:349–56. 31090576 10.1097/MOP.0000000000000756

[19] Chan KH, Stark JM, Mosquera RA, et al. Screening for asthma in preschool children with sickle cell disease. J Asthma. 2023;60:1787–92. 36867136 10.1080/02770903.2023.2187305

[20] Yahouédéhou SCMA, da Guarda CC, Figueiredo CVB, et al. Hydroxyurea alters hematological, biochemical and inflammatory biomarkers in Brazilian children with SCA: investigating associations with βS haplotype and α-thalassemia. PLoS ONE. 2019;14:e0218040. 31306416 10.1371/journal.pone.0218040 PMC6629070

[21] Rankine-Mullings AE, Nevitt SJ. Hydroxyurea (hydroxycarbamide) for sickle cell disease. Cochrane Database Syst Rev. 2022;9:CD002202. 36047926 10.1002/14651858.CD002202.pub3 PMC9435593

